# Supplementary material for: Relationship Between the Presence of Red Complex Species and the Distribution of Other Oral Bacteria, Including Major Periodontal Pathogens in Older Japanese Individuals
Source: Int J Mol Sci. 2024 Nov 14;25(22):12243. doi: 10.3390/ijms252212243 (PMC11594330; doi:10.3390/ijms252212243)
Supplement: Supplementary file 1 [file ijms-25-12243-s001.zip › ijms-3271776-supplementary.pdf]

Supplementary Table S1. Distribution of subjects' tooth numbers.

Number of teeth	*Pg*-positive (n=85)	*Td*-positive (n=16)	*Tf*-positive (n=88)	All red complex-positive (n=13)	No red complex-positive (n=10)	Total (n=116)
0-9	8 (9.4%)	1 (6.3%)	9 (10.2%)	1 (7.7%)	1 (10.0%)	12 (10.3%)
10-19	16 (18.8%)	2 (12.5%)	16 (18.2%)	2 (15.4%)	20 (20.0%)	20 (17.2%)
20-	61 (71.8%)	13 (81.3%)	63 (71.6%)	10 (76.9%)	7 (70.0%)	84 (72.4%)

Supplementary Table S2. Multivariate analysis in the presence or absence of *Campylobacter rectus*.

	Model 1				Model 2	
Variables	Odds ratio (95% confidence interval)	*P* value	Odds ratio (95% confidence interval)	*P* value	Odds ratio (95% confidence interval)	*P* value
Age	1.00 (0.91-1.11)	0.983	1.03 (0.93-1.14)	0.536	1.03 (0.93-1.15)	0.536
Sex	0.94 (0.33-2.69)	0.903	0.91 (0.30-2.79)	0.873	0.91 (0.30-2.79)	0.873
Teeth number	1.05 (0.98-1.12)	0.169	1.07 (0.99-1.15)	0.064	1.07 (0.99-1.15)	0.069
Ec	**4.37 (1.49-12.8)**	**0.007**	-	-	**3.70 (1.20-11.4)**	**0.022**
Co	-	-	**7.08 (2.05-24.40)**	**0.002**	**5.65 (1.58-20.2)**	**0.008**

Bold values indicate statistical significance at $P < 0.05$. *Ec*; *Eikenella corrodens*, *Co*; *Capnocytophaga ochracea*.

Supplementary Table S3. Multivariate analysis in the presence or absence of *Prevotella nigrescens*.

	Model 1				Model 2	
Variables	Odds ratio (95% confidence interval)	*P* value	Odds ratio (95% confidence interval)	*P* value	Odds ratio (95% confidence interval)	*P* value
Age	1.01 (0.91-1.13)	0.770	1.05 (0.94-1.19)	0.386	1.04 (0.93-1.17)	0.473
Sex	1.04 (0.32-3.40)	0.944	1.39 (0.43-4.47)	0.583	0.98 (0.28-3.36)	0.971
Teeth number	0.99 (0.91-1.07)	0.792	1.00 (0.92-1.10)	0.944	1.00 (0.92-1.09)	0.963
Ec	**4.10 (1.18-14.2)**	**0.026**	-	-	3.53 (0.98-12.7)	0.054
Co	-	-	**4.99 (1.34-18.60)**	**0.017**	**4.03 (1.02-15.8)**	**0.045**

Bold values indicate statistical significance at $P < 0.05$. Bold values indicate statistical significance at $P < 0.05$. *Ec*; *Eikenella corrodens*, *Co*; *Capnocytophaga ochracea*.

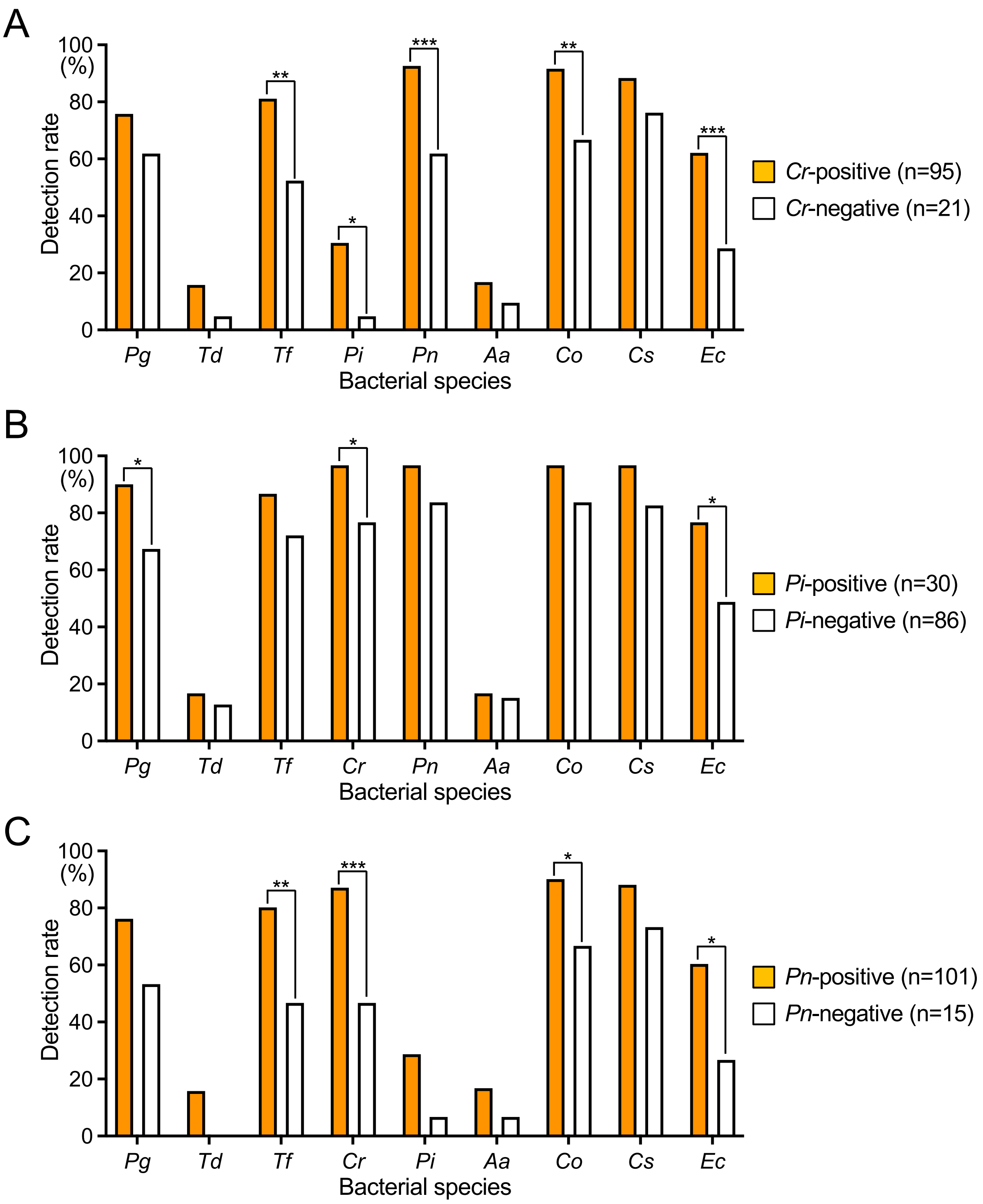

Supplementary Fig. S1. Distribution of each periodontopathic bacterial species with or without orange complex. Distribution of each periodontopathic bacterial species in *Campylobacter rectus*-positive and *C. rectus*-negative (A), *Prevotella intermedia*-positive and *P. intermedia*-negative (B), *Prevotella nigrescens*-positive and *P. nigrescens*-negative (C). *P<0.05, **P<0.01, ***P<0.001 between each group. *Cr*; *C. rectus*, *Pi*; *P. intermedia*, *Pn*; *P. nigrescens.*

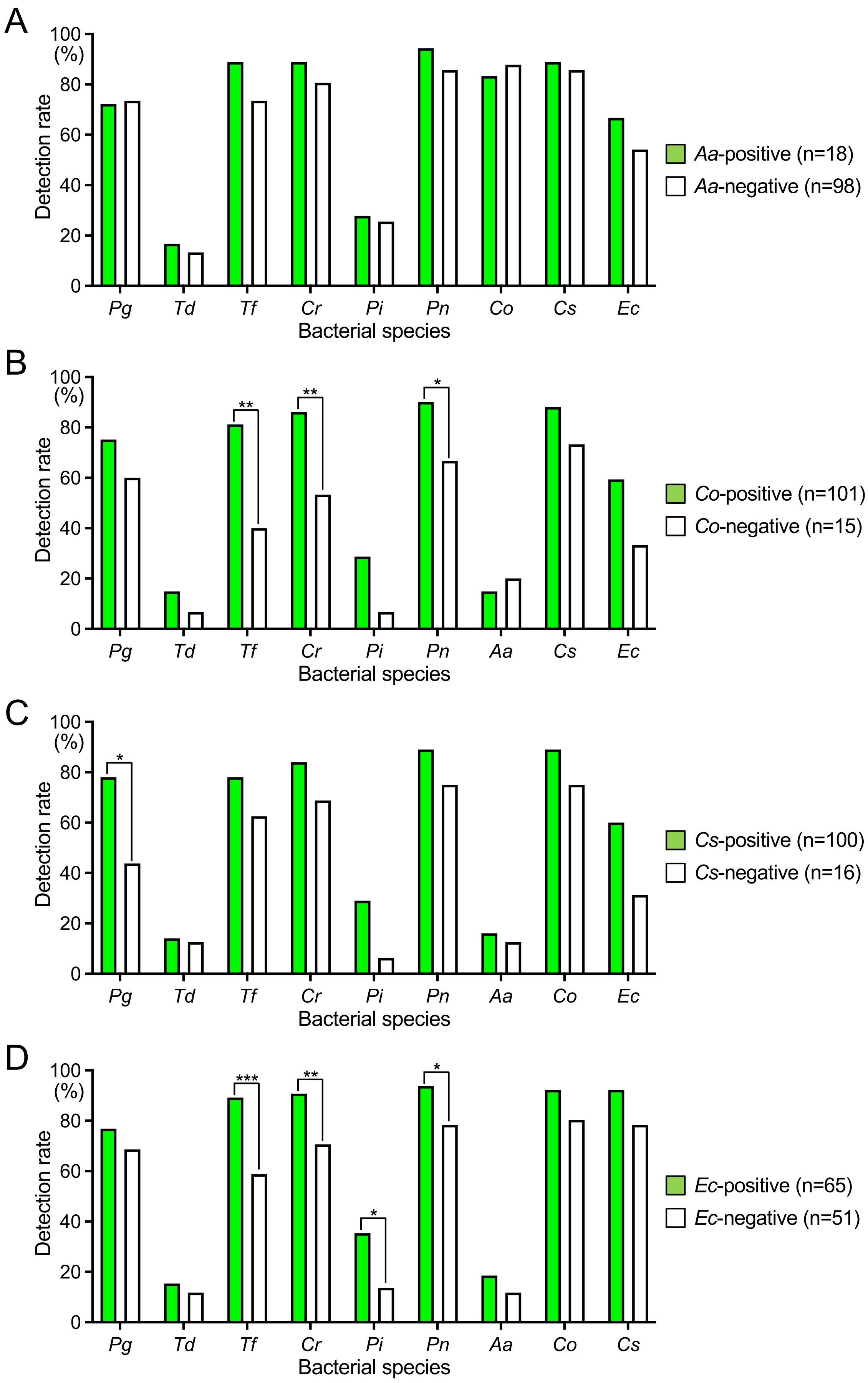

Supplementary Fig. S2. Distribution of each periodontopathic bacterial species with or without green complex. Distribution of each periodontopathic bacterial species in *Aggregatibacter actinomycetemcomitans* -positive and *A. actinomycetemcomitans* -negative (B), *Capnocytophaga ochracea* -positive and *C. ochracea* -negative (C), *Capnocytophaga sputigen* -positive and *C. sputigen* -negative (D), *Eikenella corrodens* -positive and *E. corrodens* -negative (E). **P*<0.05, ***P*<0.01, ****P*<0.001 between each group. *Aa*; *A. actinomycetemcomitans*, *Co*; *C. ochracea*, *Cs*; *C. sputigena*, and *Ec*; *E. corrodens*.
